# Context-specific and context-invariant computations of interval timing

**DOI:** 10.3389/fnins.2023.1249502

**Published:** 2023-09-20

**Authors:** Ahmad Pourmohammadi, Mehdi Sanayei

**Affiliations:** ^1^School of Cognitive Sciences, Institute for Research in Fundamental Sciences (IPM), Tehran, Iran; ^2^Center for Translational Neuroscience (CTN), Isfahan University of Medical Sciences, Isfahan, Iran

**Keywords:** interval timing, explicit timing, sensory timing, motor timing, Bayesian inference

## Abstract

**Introduction:**

An accurate sense of time is crucial in flexible sensorimotor control and other cognitive functions. However, it remains unknown how multiple timing computations in different contexts interact to shape our behavior.

**Methods:**

We asked 41 healthy human subjects to perform timing tasks that differed in the sensorimotor domain (sensory timing vs. motor timing) and effector (hand vs. saccadic eye movement). To understand how these different behavioral contexts contribute to timing behavior, we applied a three-stage Bayesian model to behavioral data.

**Results:**

Our results demonstrate that the Bayesian model for each effector could not describe bias in the other effector. Similarly, in each task the model-predicted data could not describe bias in the other task. These findings suggest that the measurement stage of interval timing is context-specific in the sensorimotor and effector domains. We also showed that temporal precision is context-invariant in the effector domain, unlike temporal accuracy.

**Discussion:**

This combination of context-specific and context-invariant computations across sensorimotor and effector domains suggests overlapping and distributed computations as the underlying mechanism of timing in different contexts.

## 1. Introduction

How the brain perceives time has been the focus of many studies over the past decades (Tsao et al., 2022). The computational goal for measuring time at the present moment is to accurately and precisely track elapsed time within an ongoing interval. This computation is integral to sensorimotor control and decision-making, yet our subjective experience of time can be biased in different contexts. A classic example of this contextual calibration is Vierordt’s law, also known as the central tendency effect (Lejeune and Wearden, 2009; Glasauer and Shi, 2021). Vierordt showed that short temporal durations tend to be overestimated in a temporal reproduction task, whereas long durations tend to be underestimated (Lejeune and Wearden, 2009). However, the underlying mechanism of these observations remains unexplained for more than a century. The central tendency effect is related to another feature of interval timing: Weber’s law. According to Weber’s law, the variability of temporal performance increases with the mean of the time interval, also known as scalar variability (Gibbon et al., 1997; Rakitin et al., 1998; Wearden and Bray, 2001; Brannon et al., 2008). Although information processing models based on the internal clock, memory-mixing, and internal noise were applied to explain contextual calibration and Weber’s law (for review see Gibbon et al., 1997; Allman et al., 2014), these approaches did not provide a quantitative prediction of the factors that contribute to contextual calibration. Furthermore, how the nervous system uses contextual calibration to improve timing behavior remains unknown.

To address the effects of contextual calibration on interval timing, a prior study used a Bayesian framework. Jazayeri and Shadlen (2010) developed and compared three probabilistic observer models with different strategies (i.e., maximum-likelihood estimation, maximum a posteriori, and Bayes least-squares). They showed that observers used the Bayes least-squares strategy to reproduce temporal intervals. This finding was concluded based on the success of the Bayes least-squares model which provided an accurate description of behavioral data in a temporal reproduction task. The Bayesian framework suggests that the observer uses two sources of information to estimate a sample interval: sensory measurements (i.e., likelihood function) and the prior knowledge of the statistical distribution. Bayesian models have made great advances in describing a variety of cognitive functions including interval timing (Shi et al., 2013; Sadibolova and Terhune, 2022). This approach proposes that an optimal observer combines noisy sensory measurements with the prior knowledge of the statistical distribution of the stimulus to improve behavior. This behavioral computation is interconnected with the trade-off between accuracy and precision: the prior-dependent bias increases for less reliable measurements. Previous studies used Bayesian models to elucidate how prior knowledge is formed in different behavioral contexts (Nagai et al., 2012; Petzschner et al., 2012; Gekas et al., 2013; Kerrigan and Adams, 2013). They suggested that representation of prior knowledge is sensory specific after extended training. Roach et al. (2017) extended this approach to interval timing. In a temporal reproduction task, they applied a Bayesian model to study how priors are learned and how they are generalized to different behavioral contexts. They showed that participants formed a single prior by generalizing across intervals coupled with different sensory modalities in early sessions. However, prior generalization was not occurred and participants formed multiple, and separate, priors across duration distributions when coupled with different effectors. The authors suggested that representation of prior knowledge is effector specific, but not sensory specific, in the temporal reproduction task without extended training. However, it remained unclear how behavioral context contribute to timing computations.

To understand the underlying mechanisms of timing, previous studies compared timing behavior in different contexts. Several psychophysical studies showed that timing behavior, especially scalar variability, is similar for different explicit timing tasks or effectors (Treisman, 1963; Keele et al., 1985; Ivry and Hazeltine, 1995; Gibbon et al., 1997; Meegan et al., 2000; Bartolo and Merchant, 2009). These results may suggest that the brain has a specialized set of circuits for measuring time across sensorimotor domains and effectors (Ivry and Schlerf, 2008). However, electrophysiological and computational studies showed that time can be encoded through changes in neural population activity over time, or population state dynamics (Buonomano and Mauk, 1994; Gouvêa et al., 2015; Egger et al., 2020; Zhou et al., 2022). Although dedicated and intrinsic models of timing, are not mutually exclusive (Merchant et al., 2008; Polti et al., 2022), converging data from psychophysical, neuroimaging and electrophysiological studies supported partially overlapping distributed timing mechanisms for interval timing (for a review see Coull and Nobre, 2008; Paton and Buonomano, 2018; Tsao et al., 2022). Indeed, a longstanding question is how these distributed timing circuits interact to shape temporal behavior. Also, it remains unknown how sensorimotor domain or motor response type affect computations about time. To address these questions, we applied and compared Bayesian models in different temporal contexts. Interval timing tasks in this study differed in either the sensorimotor domain (sensory timing vs. motor timing) and effector (hand vs. saccadic eye movement). Sensory timing refers to tasks in which decisions are based on the temporal structure of events while motor timing refers to tasks in which subjects time their own action. We hypothesized that temporal accuracy and precision are context-invariant if there are similar computations across different contexts, but if the brain uses several different temporal computations in different contexts, we would expect to observe context-specific results. We also would like to investigate how these different behavioral contexts affect each stage of computations about measuring time.

## 2. Materials and methods

### 2.1. Subjects

We enrolled 41 healthy human subjects, (22 females, 26.76 ± 4.30 years old, reported as mean ± sd). All participants were informed of the general purpose of the study but were naïve about the scientific questions and tasks, except for two who were the authors. Subjects had normal or corrected-to-normal vision and no history of neurological or psychiatric disorders. Written informed consent was obtained from all participants before the start of the study. Subjects performed three psychophysics tasks, each task with two different motor response types in separate sessions. Here we only report results from two of those tasks. We enrolled the same subjects in all tasks, and the order of tasks was counterbalanced between subjects. The Ethics Committee of the Institute for Research in Fundamental Sciences (IPM) approved this study.

### 2.2. Apparatus

The experiments were carried out on a computer running Linux operating system, on MATLAB (2016b), with Psychtoolbox 3 extension (Brainard, 1997; Pelli, 1997; Kleiner et al., 2007). Stimuli were presented on a monitor (17″) placed ~57 cm from the subject with a 60 Hz refresh rate. The subject sat comfortably on a chair in a dimly lit room to participate in this study, with the head stabilized by a head and chin rest. An EyeLink 1000 infrared eye tracking system (SR Research, Mississauga, Ontario) was used to record eye movements at 1 kHz.

### 2.3. Temporal reproduction task

Each trial began with the presentation of a central fixation point (diameter: 0.2°) and two peripheral targets (left target: 0.5° diameters, right target: 2° diameter, 10° eccentricity). After the subject acquired fixation within a ±1° of fixation point, a trial would start. After a random delay (500 ms plus a random sample from an exponential distribution with a mean of 250 ms), two similar wheel-like stimuli (2.5° diameter) were flashed (for 26.6 ms each) sequentially around the fixation point. The presented stimuli were a circle consisting of 6 sectors of equal size. The sectors were colored yellow (150 150 50 in RGB space) and purple (150 50 150), three each. The subject measured the time from the beginning of the first flash to the beginning of the second flash, sample interval or *t*_s_, and produced a matching interval, *t*_r_, by pressing the right arrow key or making a saccade to the right target, depending on the block. Across trials, *t*_s_ was sampled from one of 5 discrete values pseudorandomly (400, 500, 700, 1,100, 1900 ms, uniform distributions). At the end of each trial, we showed the response error (*t*_r_ − *t*_s_) as feedback for 0.8 s to the subject. The inter-trial interval was 1.2 s, and each block contained 40 trials. Each subject participated in 6 blocks. In 3 blocks, they responded with their hand, and in 3 blocks, they responded with a saccadic eye movement. The order of response type was counterbalanced between subjects.

### 2.4. Temporal discrimination task

**Figure 1:**
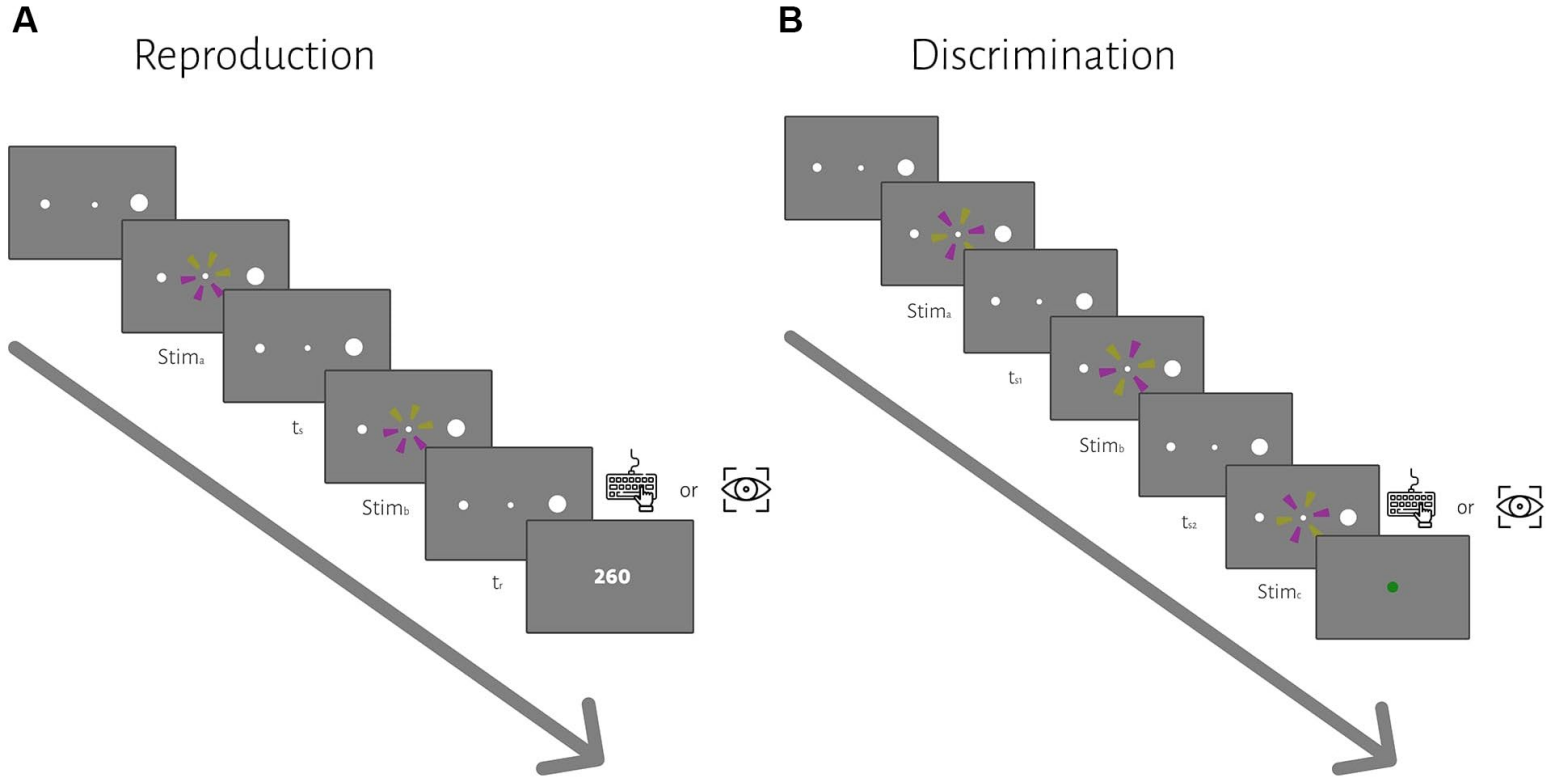
The sequence of trial events for the reproduction **(A)** and discrimination **(B)** tasks. **(A)** In the reproduction task, subjects had to measure and reproduce sample intervals, *t*_s_. After the subject acquired fixation and a random delay, *t*_s_ was demarcated by two visual flashes (stim_a_ and stim_b_). At the end of each trial, we showed the response error (*t*_r_ − *t*_s_) as feedback. **(B)** In the discrimination task, subjects had to measure and to compare two different sample intervals, *t*_s1_ and *t*_s2_, demarcated by three visual flashes (stim_a_, stim_b_, and stim_c_). *t*_s1_ were selected from a discrete uniform distribution, same as the *t*_s_ in the reproduction task. Subjects compared *t*_s2_ with *t*_s1_ and chose whether *t*_s2_ was longer or shorter than *t*_s1_ via a manual key press or saccadic eye movement in different blocks. At the end of each trial, visual feedback was presented to the subject.

Each trial started with the presentation of a fixation point and two peripheral targets (the same as the reproduction task). After a random delay, the first interval (*t*_s1_) started with the presentation of the first stimulus and ended with the presentation of the second stimulus (Figure 1B). The second interval (*t*_s2_) immediately started with the second stimulus and ended with the presentation of the third stimulus. The subject measured the time between the beginning of successive flashes as described in the reproduction task, *t*_s1_ and *t*_s2_. The stimuli were the same as the ones we used in the reproduction task, but the order of yellow and purple sectors was different. During the *t*_s1_ or *t*_s2_ interval, only the fixation point was shown (i.e., empty interval). The subject had to compare *t*_s2_ with *t*_s1_; If *t*_s2_ was longer than *t*_s1,_ then they had to press the right arrow key or make a saccade to the right target, if *t*_s2_ was shorter than *t*_s1,_ then they had to press the left arrow key or made a saccade to the left target. Across trials, *t*_s1_ was sampled from the same 5 discrete values as in the reproduction task. Duration of *t*_s2_ was *t*_s1_ duration ± [6, 12, 24, 48]% of *t*_s1_ duration, selected pseudorandomly on each trial. Feedback was shown at the end of each trial for 0.8 s (a green circle with a 1.25° diameter for correct trials and a red circle of the same size for incorrect trials). The inter-trial interval was 1.2 s, and each block contained 40 trials. Each subject participated in 12 blocks, half with hand response and the other half with eye response. The order of response type was counterbalanced between subjects.

### 2.5. Analysis of behavioral data

In the reproduction task, we excluded outlier *t*_r_ which identified with Interquartile range method (for each subject; 166 from 10,080 trials in total). The mean, standard deviation and range of *t*_r_ was calculated for hand and eye blocks (Table 1). We plotted mean of reproduction time (*t*_r_) as a function of interval duration (*t*_s_) for each subject and fitted a linear regression function to them. The fitted regression line yielded the magnitude of the compressive bias [*C* = 1—the slope of the regression (Roach et al., 2017)] and the indifference point [IP, as the time value at which the fitted regression line intersects the diagonal unity line (Roach et al., 2017)]. We also evaluated the relationship between the interval duration (*t*_s1_^2^) and timing variance (*σ*^2^) by fitting another linear regression, according to Weber’s law. The resulting slope and intercept correspond to the time-dependent (Weber’s fraction) and time-independent processes, respectively (Merchant et al., 2008). These analyses were done separately for blocks that subject responded by hand and saccadic eye movement.

**Table 1:** The mean, standard deviation, and range of tr for each effector and interval.

Effector	Intervals	Mean ± std	Min–max
Hand	0.4	0.53 ± 0.12	0.23–1.38
	0.5	0.59 ± 0.14	0.26–1.57
	0.7	0.78 ± 0.17	0.08–1.79
	1.1	1.07 ± 0.23	0.24–3.74
	1.9	1.56 ± 0.35	0.05–3.58
Eye	0.4	0.49 ± 0.14	0.22–1.22
	0.5	0.55 ± 0.18	0.24–2.66
	0.7	0.72 ± 0.22	0.24–1.79
	1.1	1.02 ± 0.28	0.25–2.12
	1.9	1.52 ± 0.4	0.23–3.01

Values are reported as mean ± std.

In the discrimination task, psychometric curves were generated by plotting the proportion of long responses over conditions (*t*_s2_) for each interval (*t*_s1_). These points were then fitted by a Gaussian cumulative density function with the non-linear least squares method. The mean (*μ*) of the fitted curve provided the point of subjective equality (PSE; the time value at which subjects judged *t*_s2_ was equal to *t*_s1_). In order to evaluate how well the psychometric function fitted with data, we measured the goodness-of-fit. We excluded poorly fitted conditions (*R* ≤ 0.50, 57 from 410 conditions in total). Subjects with more than two excluded conditions were excluded entirely from all analyses (4 subjects). We characterized the relationship between interval duration (*t*_s1_) and PSE by fitting a linear regression. The results of this analysis yielded C and IP, as described before. We also evaluated the relationship between the interval duration (*t*_s1_^2^) and variance (*σ*^2^) of the fitted Gaussian cumulative density function by fitting another linear regression to calculate Weber’s parameters. These analyses were performed separately for blocks in which subjects responded by hand or saccadic eye movement.

We excluded subjects if the calculated indifference point was outside the range of presented intervals (i.e., shorter than 400 and longer than 1900 ms) in the reproduction task (4 subjects) or the discrimination task (4 subjects, one was shared with exclusion based on the goodness-of-fit). Based on these exclusion criteria, 11 subjects were excluded from all analyses, and 30 remained.

To examine whether these computed variables were different between sensorimotor domains and between effectors, for each variable, we performed a two-way repeated measure ANOVA. Wherever we found a significant interaction between sensorimotor domain and effector, we applied HSD Tukey statistical test to control for multiple comparisons.

### 2.6. The Bayesian observer model

**Figure 2:**
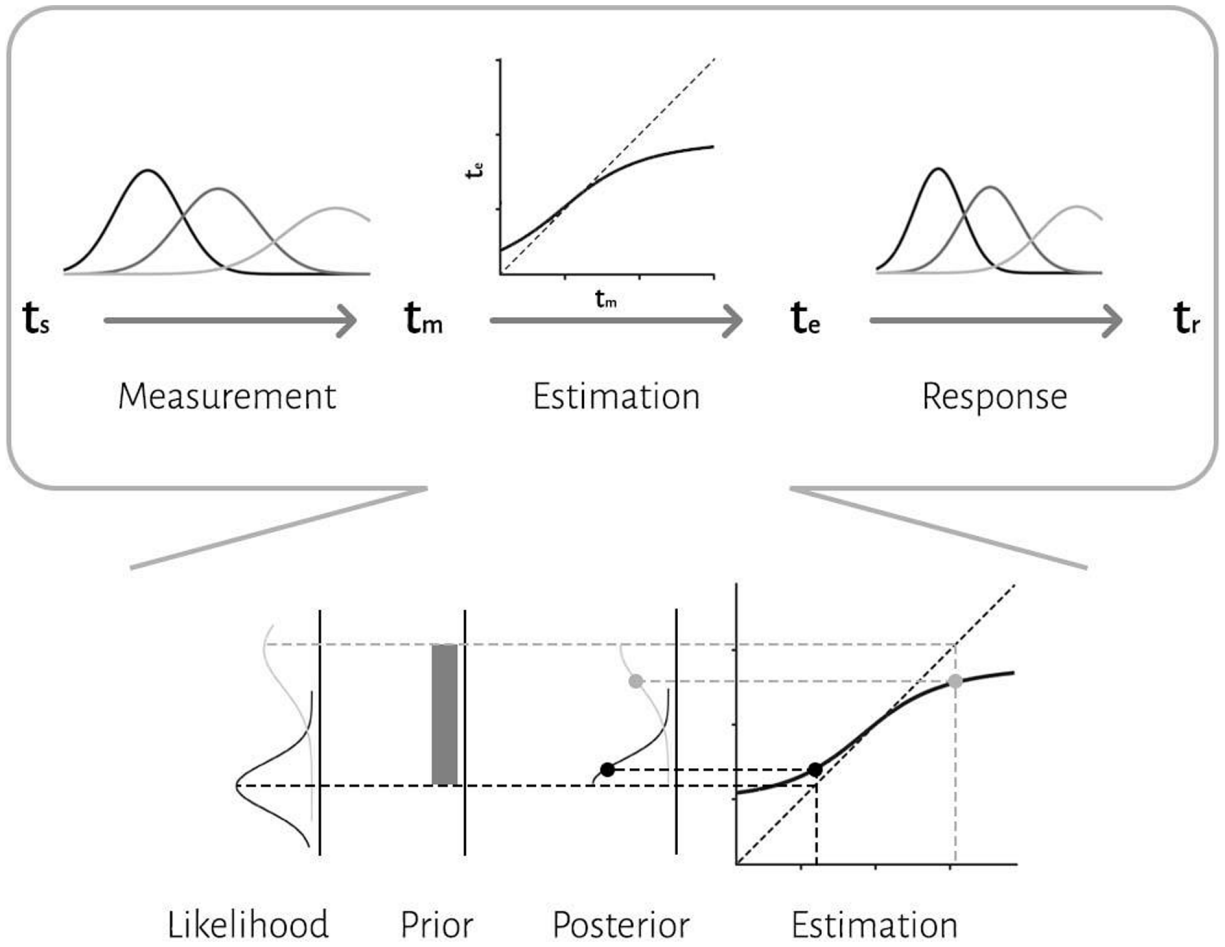
The three-stage architecture of the Bayesian observer model. In the first stage, the observer takes noisy measurements, tm, from sample intervals, *t*_s_. Measurement noise was modeled as a Gaussian function. The second stage is a Bayes least-squares (BLS) estimator. The estimator is a deterministic function, *f*(*t*_m_), that maps *t*_m_ to *t*_e_. In the third stage, the observer uses *t*_e_ to respond, *t*_r_. The relationship between *t*_r_ and *t*_e_ is characterized by motor noise, which was modeled by a Gaussian distribution. An illustration of how compressive biases arise in the model is depicted at the bottom. According to the Bayesian approach, an observer combines noisy sensory measurements (i.e., likelihood) with the prior knowledge of the statistical distribution of the stimulus to improve behavior.

We applied a Bayesian model with three stages (Jazayeri and Shadlen, 2010): measurement, estimation and motor response (Figure 2). In the first stage, an observer takes noisy measurements, *t*_m_, from sample intervals, *t*_s._ Measurement noise was modeled as a Gaussian function. We modeled the measurement stage as a Gaussian distribution with mean *t*_s_ and standard deviation *w*_m_*t*_s_, whereby the standard deviation grows as a constant fraction (*w*_m_) of the mean. This stage is also known as the likelihood function, *λ*:


$$\lambda_{t_{m}}\left(t_{s}\right)=p\left(t_{m}|t_{s}\right)=\frac{1}{\sqrt{2\pi {\left(w_{m}t_{s}\right)}^{2}}}\;e^{\frac{-{\left(t_{s}-t_{m}\right)}^{2}}{2{\left(w_{m}t_{s}\right)}^{2}}}$$

In the second stage, the Bayesian model combines the likelihood function and prior and use mean of the posterior to map the resulting posterior probability distribution onto an estimate, *t*_e_. Bayes least-squares (BLS) was used as the mapping rule in our model.


$$f\left(t_{m}\right)=\frac{\int_{t_{s}^{\text{min}}}^{t_{s}^{\text{max}}}t_{s}p\left(t_{m}|t_{s}\right)dt_{s}}{\int_{t_{s}^{\text{min}}}^{t_{s}^{\text{max}}}p\left(t_{m}|t_{s}\right)dt_{s}}$$

In the third stage, the ideal observer uses *t*_e_ to respond, *t*_r_. The relationship between *t*_r_ and *t*_e_ is characterized by motor noise, which was modeled by a Gaussian distribution with mean *t*_e_ and standard deviation *w*_r_*t*_e_.


$$p\left(t_{r}|t_{e}\right)=\frac{1}{\sqrt{2\pi {\left(w_{r}t_{e}\right)}^{2}}}\;e^{\frac{-{\left(t_{r}-t_{e}\right)}^{2}}{2{\left(w_{r}t_{e}\right)}^{2}}}$$

In the reproduction task, our psychophysical data consisted of pairs of sample intervals and reproduction times (*t*_s_ and *t*_r_). We derived a direct relationship between reproduction times and sample intervals in the Bayesian model (Jazayeri and Shadlen, 2010; Acerbi et al., 2012). This formulation was then used to describe subject’s responses in each effector, separately (Jazayeri and Shadlen, 2010; Acerbi et al., 2012). In the discrimination task, we transformed the psychometric function (Gaussian cumulative function) to response distribution (Gaussian probability function) with same parameters, as previous studies described (Getty, 1975; Keele et al., 1985; Merchant et al., 2008). Responses were generated from this distribution and we had pairs of sample intervals and responses. Then same Bayesian formulation was used to describe subjects’ responses for each effector:


$$p\left(t_{r}|t_{s},w_{m},w_{r}\right)=\int p\left(t_{r}|f\left(t_{m}\right),w_{r}\right)p\left(t_{m}|t_{s},w_{m}\right)dt_{m}$$

We maximized the likelihood of model parameters *w*_m_ and *w*_r_ across all *t*_s_ and *t*_r_ values. Maximum likelihood estimation was performed with minimize function in SciPy library, using the Nelder–Mead downhill simplex optimization method. We evaluated the success of the fitting procedure by repeating the search with several different initial values.

We performed Pearson correlation between subjects’ data and Bayesian model predicted data. To counteract the multiple comparisons problem, we performed Bonferroni correction. We showed the effect sizes using partial eta squared (*η*^2^) in the ANOVA and Bayes factor (BF) in the correlation analyses. Statistical analysis was performed with Python 3.8.5. A significant level was defined as *p*-value less than 0.05.

## 3. Results

In this study, we asked how sensorimotor domain or effector affects computations about time. To answer this question, first we compared behavioral data between different sensorimotor domains and different effectors.

**Figure 3:**
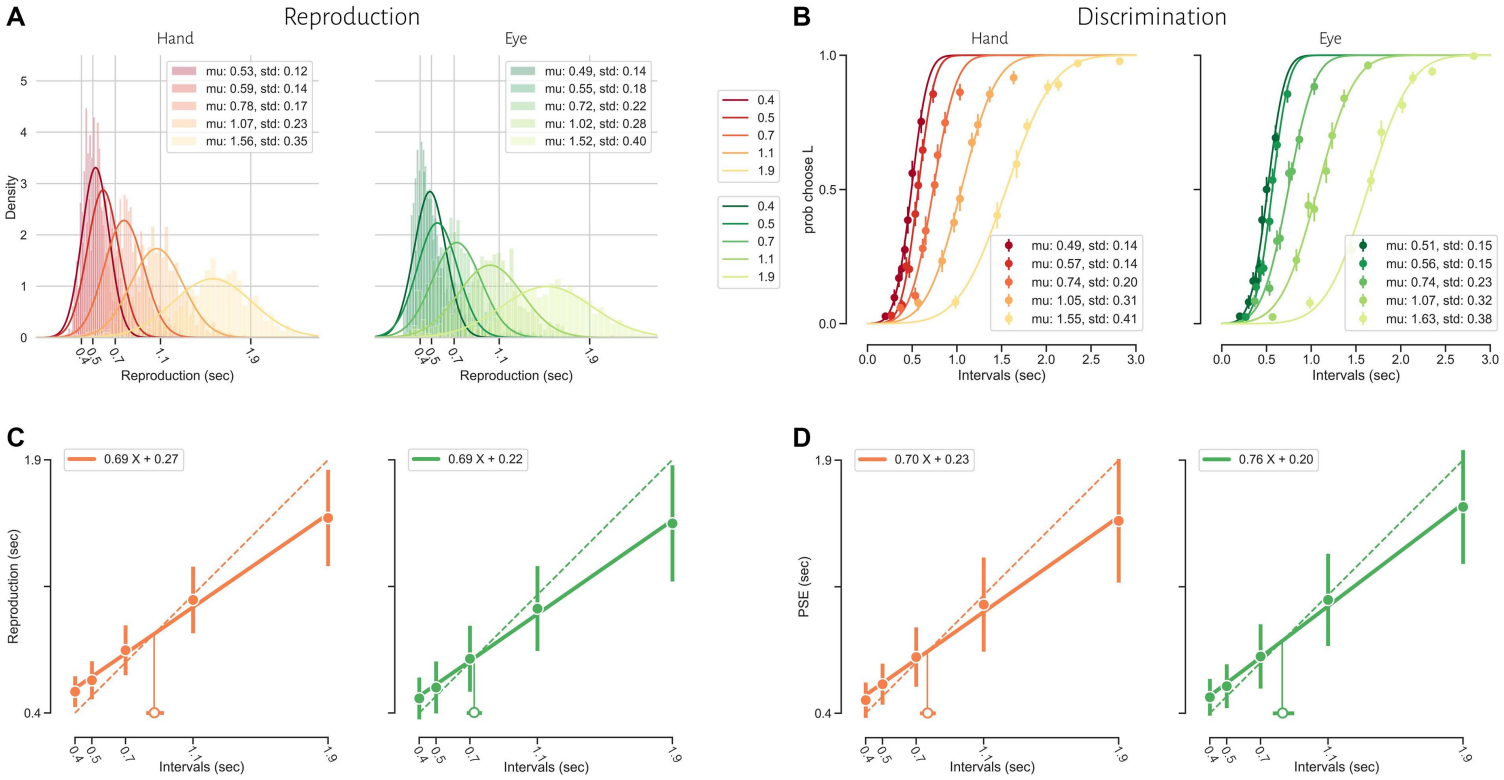
Timing behavior in the reproduction (**A** and **C**) and discrimination (**B** and **D**) tasks. **(A)** The distribution of reproduction times. **(B)** The proportion of long responses over conditions (*t*_s2_) for each interval (*t*_s1_). **(C)** Mean reproduction time as a function of interval duration in the reproduction task (filled symbols). Error bars show standard deviation of reproduction time. Solid lines show best-fitting linear regression function, whereas the dotted diagonal lines denote unbiased performance. Open symbol represents the estimated indifference point along with bootstrapped 95% confidence intervals. **(D)** Point of subjective equality (PSE) as a function of interval duration in the discrimination task. Error bars show standard deviation of fitted psychometric function.

**Figure 4:**
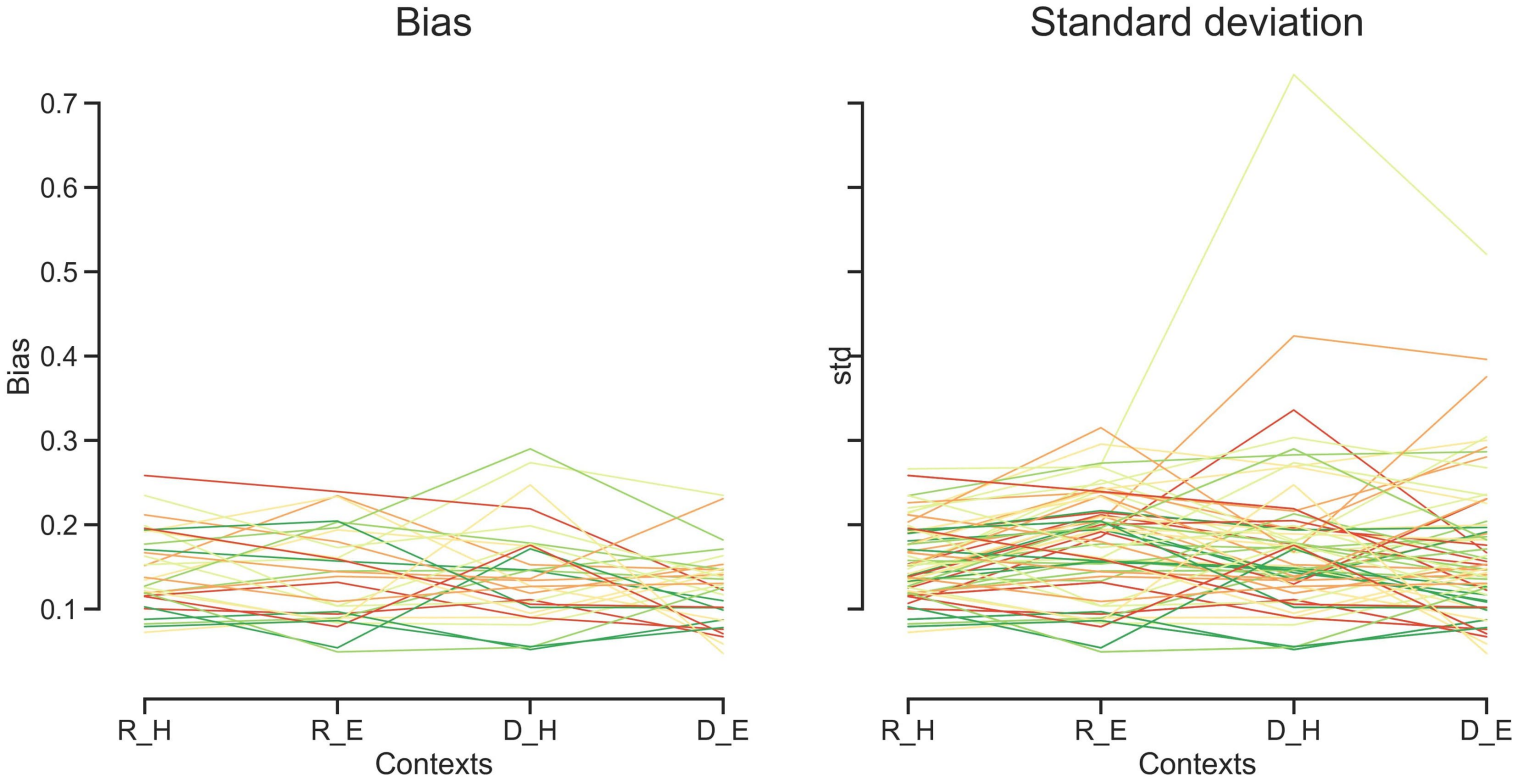
Bias and standard deviation for each subject over different tasks and effectors. Each line represents one subject. Discrimination task (D), reproduction task (R), hand (H), eye (E).

Human subjects were asked to perform two different timing tasks and report their choices via a button press or eye movement (Figure 1). Subjects showed both characteristic features of interval timing: central tendency bias and scalar variability (Figure 3). First, response intervals (*t*_r_, mean of reproduction time and point of subjective equality for reproduction and discrimination, respectively) were systematically biased toward the mean of the presented distribution, a phenomenon known as central tendency bias. We quantified this central tendency bias with two parameters of a regression analysis (see Methods): the magnitude of the compressive bias and the indifference point. Second, the measurement of longer sample intervals engenders more uncertainty, a phenomenon known as scalar variability, as can be seen in increasing the standard deviation (*σ*) as interval time increased. Scalar variability was quantified with two parameters of Weber’s law (see Method), the time-dependent (Weber’s fraction) and time-independent parameters of Weber’s law. We also showed temporal bias and standard deviation for each subject in different tasks and effectors (Figure 4).

To examine the effects of sensorimotor domain and effector on temporal processing, we compared central tendency bias and scalar variability between different sensorimotor domains and effectors. We did not find any significant difference between blocks employing key presses or saccadic eye movements when we compared compression bias (*F* = 2.85, *p* = 0.1, *η*^2^ = 0.01), indifference point (*F* = 0.55, *p* = 0.44, *η*^2^ < 0.01), Weber’s fraction (*F* = 0.00, *p* = 0.99, *η*^2^ < 0.01) and time-independent parameter (*F* = 3.14, *p* = 0.09, *η*^2^ = 0.03). Similarly, we did not find any significant difference between reproduction and discrimination blocks when we compared compression bias (*F* = 1.78, *p* = 0.19, *η*^2^ = 0.02), indifference point (*F* = 0.35, *p* = 0.58, *η*^2^ < 0.01), Weber’s fraction (*F* = 1.66, *p* = 0.21, *η*^2^ = 0.02) and time-independent parameter (*F* = 2.10, *p* = 0.16, *η*^2^ = 0.02). The interaction of effector and task on the indifference point was significant (*F* = 12.32, *p* < 0.01, *η*^2^ = 0.01) but further analysis using HSD Tukey test did not find any significant effect in multiple comparison between groups (Table 2).

**Table 2:** Timing behavior between sensorimotor domains and between effectors.

	Reproduction		Discrimination		ANOVA
	Hand	Eye	Hand	Eye	
C	0.31 ± 0.13	0.31 ± 0.12	0.30 ± 0.14	0.25 ± 0.10	Effector: *F* = 2.85, *p* = 0.10, *η*^2^ = 0.01
					Task: *F* = 1.78, *p* = 0.19, *η*^2^ = 0.02
					Effector × task: *F* = 1.59, *p* = 0.22, *η*^2^ = 0.01
IP	0.92 ± 0.24	0.75 ± 0.20	0.82 ± 0.26	0.92 ± 0.38	Effector: *F* = 0.55, *p* = 0.44, *η*^2^ < 0.01
					Task: *F* = 0.35, *p* = 0.58, *η*^2^ < 0.01
					**Effector** × **task: *F* = 12.32, *p* < 0.001**^a^**, *η***^**2**^ **= 0.06**
Web_s	0.023 ± 0.023	0.031 ± 0.020	0.047 ± 0.090	0.040 ± 0.067	Effector: *F* = 0.00, *p* = 0.99, *η*^2^ < 0.01
					Task: *F* = 1.66, *p* = 0.21, *η*^2^ = 0.02
					Effector × task: *F* = 2.31, *p* = 0.14, *η*^2^ < 0.01
Web_i	0.010 ± 0.011	0.021 ± 0.016	0.020 ± 0.033	0.025 ± 0.027	effector: *F* = 3.14, *p* = 0.09, *η*^2^ = 0.03
					Task: *F* = 2.10, *p* = 0.16, *η*^2^ = 0.02
					Effector × task: *F* = 0.77, *p* = 0.39, *η*^2^ < 0.01
*w*_m_	0.21 ± 0.05	0.19 ± 0.03	0.18 ± 0.03	0.19 ± 0.03	Effector: *F* = 2.93, *p* = 0.1, *η*^2^ = 0.01
					**Task: *F* = 5.57, *p* = 0.03**^a^**, *η***^**2**^ **= 0.04**
					**Effector × task: *F* = 5.44, *p* < 0.03**^a^**, *η***^**2**^ **= 0.02**
*w*_r_	0.16 ± 0.05	0.24 ± 0.05	0.24 ± 0.11	0.24 ± 0.10	**Effector: *F* = 10.31, *p* < 0.01**^a^**, *η***^**2**^ **= 0.05**
					**Task: *F* = 6.03, *p* = 0.02**^a^**, *η***^**2**^ **= 0.05**
					**Effector** × **task: *F* = 19.66, *p* < 0.01**^a^**, *η***^**2**^ **= 0.06**

The magnitude of the compressive bias (C), indifference point (IP), Weber’s fraction (Web_s), time-independent parameters of Weber’s law (Web_i), measurement constant coefficient of variation (*w*_m_), response constant coefficient of variation (*w*_r_).

a Denotes a significant difference in two-way repeated measure ANOVA results.

### 3.1. The Bayesian observer model

To further evaluate the effects of sensorimotor domain and effector on temporal processing and to understand the computations from which these effects might arise, we applied a Bayesian model with three stages (i.e., measurement, estimation and motor response) and two free parameters (i.e., measurement noise (*w*_m_) and motor noise (*w*_r_); Methods, Figure 2). We fitted the parameters of the Bayesian model, *w*_m_ and *w*_r_, for each subject on the basis of *t*_r_. Then we compared *w*_m_ and *w*_r_ between different tasks and different motor response types. While effector did not have any significant effect (*F* = 2.93, *p* = 0.10, *η*^2^ = 0.01), tasks (*F* = 5.57, *p* < 0.03, *η*^2^ = 0.04) had significant effects on *w*_m_, and the interaction between effector and task reached significant level (*F* = 5.44, p < 0.03, *η*^2^ = 0.02). Post-hoc analysis showed that *w*_m_ in the discrimination with hand (0.18 ± 0.03) was significantly smaller than in the reproduction with hand (0.21 ± 0.05, adjusted *p* = 0.03). Both effector (*F* = 10.31, *p* < 0.01, *η*^2^ = 0.05) and tasks (*F* = 6.03, *p* = 0.02, *η*^2^ = 0.05) had significant effects on *w*_r_. Their interaction was significant as well (*F* = 19.66, *p* < 0.01, *η*^2^ = 0.06). Additional analysis showed that *w*_r_ was significantly smaller in the reproduction with hand (0.16 ± 0.05) than in the discrimination with hand (0.24 ± 0.11, adjusted *p* < 0.01); than the discrimination with eye (0.24 ± 0.10, adjusted *p* < 0.01); and the reproduction with eye (0.24 ± 0.05, adjusted *p* < 0.01, Table 2).

**Figure 5:**
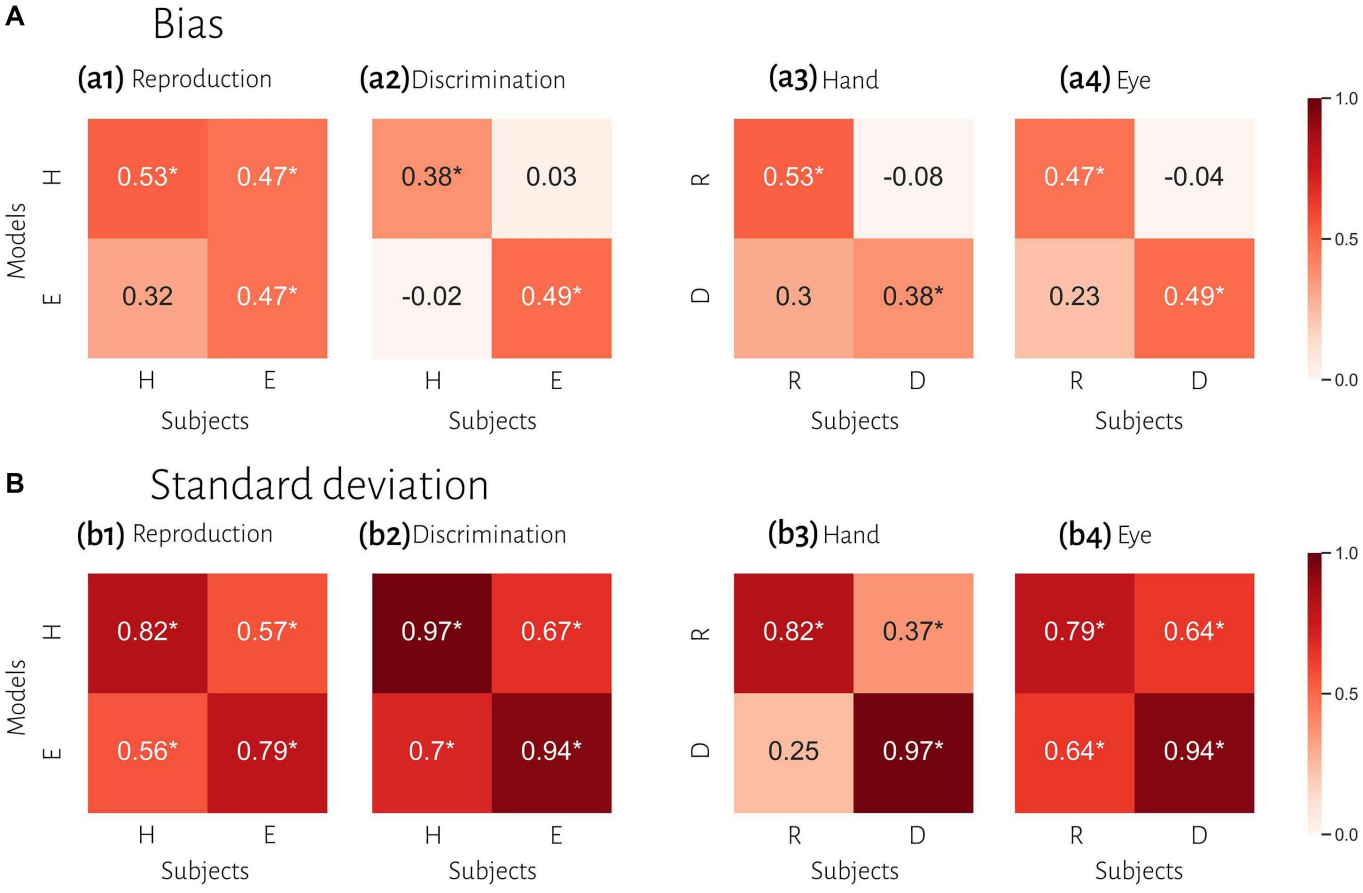
Comparison of timing behavior in human and model observers. **(A)** The correlation between subject’s bias and simulated data from best fitting model for each subject. **(B)** The correlation between subject’s variability and simulated data from best fitted model for each subject. Reproduction (R), discrimination (D), hand (H), eye (E). ^*^Denotes a significant correlation.

To compare subjects’ responses to those predicted by the model, we simulated each subject’s responses using the fitted Bayesian model and compared model predictions to the actual responses using the bias and variability statistics. The correlations showed that our Bayesian model can predict subjects’ responses in each effector and each task (Figure 5). The model performed better in the standard deviation variable compared with bias (Figure 5A vs. Figure 5B). When we performed Bonferroni correction the Bayesian model did not describe subjects’ data in the discrimination task with hand (*r* = 0.378, *p*-unc = 0.039, *p*-corr = 0.157, BF = 1.7; Table 3).

**Table 3:** Correlation between the subject’s data and model-predicted data in each context.

Subjects	Models	*r*	*p*	*p* (corr)	BF
**Bias**					
**R_H**	**R_H**	**0.527**	**0.003** ^a^	**0.011** ^a^	**16.2**
R_H	R_E	0.316	0.089	0.356	0.9
**R_E**	**R_H**	**0.471**	**0.009** ^a^	**0.035** ^a^	**6**
**R_E**	**R_E**	**0.471**	**0.009** ^a^	**0.034** ^a^	**6.1**
D_H	D_H	0.378	0.039^a^	0.157	1.7
D_H	D_E	−0.017	0.929	1	0.2
D_E	D_H	0.028	0.884	1	0.2
**D_E**	**D_E**	**0.492**	**0.006** ^a^	**0.023** ^a^	**8.6**
R_H	D_H	0.304	0.102	0.204	0.8
D_H	R_H	−0.085	0.656	1	0.2
R_E	D_E	0.232	0.217	0.434	0.4
D_E	R_E	−0.045	0.815	1	0.2
**Standard deviation**					
**R_H**	**R_H**	**0.817**	**<0.001** ^a^	**<0.001** ^a^	**>1,000**
**R_H**	**R_E**	**0.560**	**0.001** ^a^	**0.005** ^a^	**31.5**
**R_E**	**R_H**	**0.566**	**0.001** ^a^	**0.005** ^a^	**35.6**
**R_E**	**R_E**	**0.785**	**<0.001** ^a^	**<0.001** ^a^	**>1,000**
**D_H**	**D_H**	**0.97**	**<0.001** ^a^	**<0.001** ^a^	**>1,000**
**D_H**	**D_E**	**0.701**	**<0.001** ^a^	**<0.001** ^a^	**>1,000**
**D_E**	**D_H**	**0.667**	**<0.001** ^a^	**<0.001** ^a^	**517.5**
**D_E**	**D_E**	**0.940**	**<0.001** ^a^	**<0.001** ^a^	**>1,000**
R_H	D_H	0.249	0.185	0.74	0.5
D_H	R_H	0.366	0.047^a^	0.188	1.4
**R_E**	**D_E**	**0.640**	**<0.001** ^a^	**<0.001** ^a^	**230.5**
**D_E**	**R_E**	**0.639**	**<0.001** ^a^	**<0.001** ^a^	**218.4**

*p* (corr) column represent Bonferroni correction results. Bayes factor (BF), discrimination task (D), reproduction task (R), hand (H), eye (E).

a Denotes a significant correlation.

To evaluate the effect of effector, we compared subjects’ responses in blocks using a key press to model predictions in blocks using a saccadic eye movement and vice versa in the same tasks. In reproduction, bias was significantly correlated between model from hand data to the data from the eye = 0.47, *p* < 0.01, BF = 6.1. The correlation between model from eye and data from hand was not significant (*r* = 0.31, *p* = 0.09, BF = 1.0). However, in the discrimination task, bias was not significantly correlated between subjects’ responses and model predictions with different effectors (Figure 5 and Table 3). In contrast, variability was significantly correlated between subjects’ responses and model predictions with different effectors in both tasks (*r*_s_ > 0.50, *p*_s_ < 0.01).

**Figure 6:**
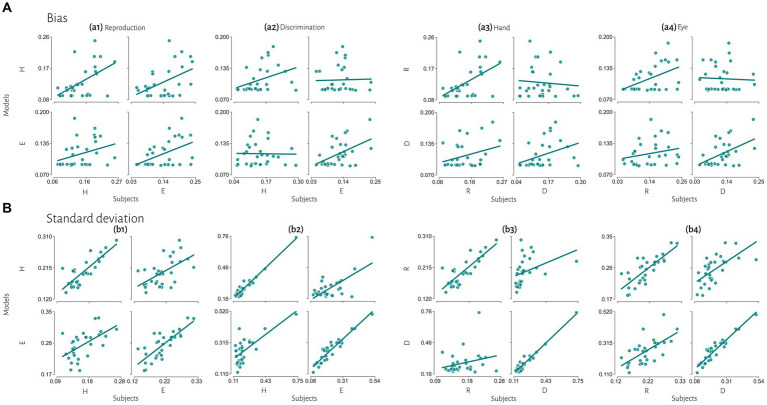
Extended comparisons of timing behavior in human and model observers. **(A)** The correlation between subject’s bias and simulated data from best fitted model for each subject. **(B)** The correlation between subject’s variability and simulated data from best fitted model for each subject. Reproduction (R), discrimination (D), hand (H), eye (E).

To evaluate the effect of tasks, we compared subjects’ responses in the reproduction task to model predictions in the discrimination task and vice versa with the same effector. Bias was not significantly correlated between subjects’ responses in each task and model fitted to the other task (Figure 5). Variability was significantly correlated between subjects’ responses and model predictions with different task in the eye blocks (model reproduction, data discrimination: *r* = 0.64, *p* < 0.01, BF = 218; model discrimination, data reproduction: *r* = 0.64, *p* < 0.01, BF = 230) but not in hand blocks (Figure 5 and Table 3). The scatter plot for each correlation analysis is shown in Figure 6.

## 4. Discussion

In this study, we investigated how behavioral contexts affect computations about time. We hypothesized that temporal accuracy and precision are context-invariant (between both tasks and effectors) if there are similar computations across different contexts, but if the brain uses several different temporal computations in different contexts, we would observe context-specific results. We found that compression bias, indifference point, and Weber’s parameters were not different between these behavioral contexts. These results might qualitatively suggest that temporal accuracy and precision are context-invariant, but this approach is not precise enough to address the mechanistic insight. We investigated further to address this question in a quantitative and predictive way. We also showed the effects of behavioral context on different stages of timing (i.e., sensing time and temporal motor response). We used the Bayesian model of interval timing (Jazayeri and Shadlen, 2010; Acerbi et al., 2012; Roach et al., 2017), which has three stages and two free parameters (i.e., measurement noise (*w*_m_) and motor noise (*w*_r_); Figure 2). Our results suggest that motor noise (*w*_r_) is context-specific in both effector and sensorimotor domains. However, measurement noise (*w*_m_) is context-specific in the sensorimotor domain, but not between effectors. In a predictive analysis, we evaluated model performance in different combination of effector and sensorimotor domains.

### 4.1. Context-specific versus context-invariant timing

Previous studies also reported that temporal precision is correlated across different sensorimotor domains (Spencer and Zelaznik, 2003) and different effectors (Keele et al., 1985). We also found similar results. Compression bias, indifference point, and Weber’s parameters were not different between these behavioral contexts. Although these observations may qualitatively suggest that subjects who are good timers in one behavioral context are also good timers in another one, converging data from psychophysical studies remain controversial (for review see Merchant et al., 2013). We used a mechanistic model to address this controversy. Our results demonstrate that the Bayesian model for each effector could not describe bias in other effector in either reproduction (Figure 5a_1_) or discrimination (Figure 5a_2_) tasks. Similarly, in each task the model predicted data could not describe bias in other task in either hand (Figure 5a_3_) or eye (Figure 5a_4_) blocks. This absence of correlation suggests that temporal accuracy is context-specific in the sensorimotor and effector domains. As we mentioned earlier, the first two stages of Bayesian model contribute to response bias. These findings and the presence of a significantly different measurement noise (*w*_m_) between different tasks suggest that timing computation in the measurement stage is different between behavioral contexts. In other words, the measurement stage is context-specific in the sensorimotor and effector domains which suggests that the brain might use different mechanisms or computations for timing when it knows that it has to engage in sensory vs. motor timing tasks with different effectors. We also found that the Bayesian model for each effector could describe standard deviation in other effector in either reproduction (Figure 5b_1_) or discrimination (Figure 5b_2_) tasks, which suggest temporal precision is context-invariant in the effector domain, unlike temporal accuracy. We found similar results in each task in eye blocks (Figure 5b_4_), but not in hand blocks (Figure 5b_3_). It seems that the temporal precision between reproduction and discrimination tasks in hand blocks (Figure 5b_3_) showed different pattern when compared with other behavioral contexts (Figures 5b_1,2,4_). Different temporal and non-temporal mechanisms might contribute to this different pattern of temporal precision between the reproduction and the discrimination tasks. Sensory and motor timing tasks have different instructions and different temporal and non-temporal computations which might be recruited in these tasks, for example in the discrimination task subject should memorize and sort two different intervals. Different working memory components between sensory and motor timing tasks may contribute to these different computations in sensorimotor domain. Another contributing factor may be inter-stimulus interval. Previous studies showed that different inter-stimulus intervals change temporal computations in the sensory timing tasks (Karmarkar and Buonomano, 2007; Sadibolova et al., 2021). Both sensory and motor timing tasks in this study did not have inter-stimulus interval (between intervals), however we cannot rule out that the lack of inter-stimulus interval has the same effect on sensory and motor timing tasks.

In sensory and motor timing, the observer’s task can be explained as accumulating evidence in the time domain and comparing it to a bound, similar to what has been shown in decision making (Simen et al., 2011). This evolving decision variable would then lead to a motor action when reaches a bound. In our sensory timing task, temporal decisions and motor responses are made by choosing between two alternatives. However, in the motor timing task, decisions and motor responses are made by choosing when to act. de Lafuente et al. (2015) used a two-alternative forced choice (2-AFC) random dots motion discrimination task showed that when decisions are communicated by different effectors neurons in the medial intraparietal (MIP) area exhibit different firing activity. Instead, decision-related activity was observed in the lateral intraparietal (LIP) area in both hand and eye blocks. We argue that at the computational level, when a temporal decision (or maybe any other decision) has to be made in a 2-AFC task, temporal measurement (the first stage in our model) raises from different computations in eye vs. hand blocks. But when an observer wants to decide when to act, a 1-AFC task, temporal measurement had the same computations between different effectors.

### 4.2. Mechanistic insight into explicit timing

In this section we are going to expand our mechanistic insight into explicit timing using the results of our study. As we discussed earlier our results showed combination of context-specific and context-invariant computations across sensorimotor and effector domains. These observations suggest overlapping and distributed computations as the underlying mechanism of timing in different contexts. Previous neuroimaging and electrophysiological studies also support this idea at the implementational level of analysis (for review see Coull and Nobre, 2008; Paton and Buonomano, 2018; Tsao et al., 2022). Several studies showed that depending on the task and timescale, many areas had been implicated in different temporal contexts (Coull et al., 2004; Jantzen et al., 2004; Bengtsson et al., 2005; Garraux et al., 2005; Jantzen et al., 2005; Pouthas et al., 2005; Jantzen et al., 2007). The results of these previous studies suggest that time is encoded in both context invariant and context specific areas. In this framework, context specific time representation can be encoded through changes in neural population activity over time in distributed neural circuits (Paton and Buonomano, 2018; Tsao et al., 2022). However, a more detailed understanding of the neural substrates of context invariant computations is lacking. These computations might be implemented in overlapping neural circuits. The effects of neuromodulatory systems on distributed neural circuits can also play a critical role in context invariant computations of time. Previous studies reported that the basal ganglia are activated in timing tasks with different effectors (Bengtsson et al., 2005), sensorimotor domains (Schubotz and von Cramon, 2001; Bueti et al., 2008), and duration scales (Jahanshahi et al., 2006). These studies suggested that context invariant computations might be implemented in the basal ganglia. Another study also reported that optogenetic manipulation of substantia nigra pars compacta (SNc) dopamine neurons can modify timing behavior (Soares et al., 2016). The results of this study suggested that dopaminergic projections from the SNc to the striatum could modify striatal population dynamics. Considering that the basal ganglia is interconnected with widespread regions of the cerebral cortex and subcortical areas, it is possible that basal ganglia can modify distributed neural circuits in timing tasks. Future studies are needed to address this question and to understand how these different timing computations are implemented in the brain.

### 4.3. Applications and future challenges

In this study we showed different context-specific and context-invariant temporal computations. Previous studies investigated interval timing in neurological and psychiatric disorders (for review see Allman and Meck, 2012). Malapani et al. (1998) studied a temporal reproduction task in patients with Parkinson’s disease and tested patients while they were ON or OFF their levodopa medication. They showed in the OFF state, temporal reproduction was impaired in both accuracy (bias) and precision (variance). Singh et al. (2021) also showed that impaired cue-evoked midfrontal ~4 Hz activity predicts increased timing variability and both timing variability and midfrontal ~4 Hz rhythms were correlated with overall cognitive impairments in patients with Parkinson’s disease. Timing tasks can be used as a proxy to reveal pathophysiological changes in different neurological and psychiatric disorders and also these studies can reveal mechanistic insights into temporal computations. Future studies can investigate different context-specific and context-invariant temporal computations in different pathophysiological conditions and expand the mechanistic understanding of these different temporal computations.

This study has some limitations. We studied explicit timing tasks and our results cannot be generalized to implicit timing tasks. We also used sensorimotor and effector (i.e., hand and eye response) domains as behavioral contexts however future studies are needed to investigate the effects of other behavioral contexts including other factors on interval timing. The effects of temporal and spatiotemporal learning (Emmons et al., 2020; Gür et al., 2023) on the contextual temporal computations are also interesting for future experiments. In this study we did not compare different computational models of interval timing. We showed that the Bayesian model can accurately describe behavioral data in different sensorimotor domains and different effectors. Our results also showed that the model capture standard deviation better than bias (Figure 5A vs. Figure 5B). We did not investigate the underlying reason of this observation, one reason might be that one parameter, *w*_m_, is responsible for catching the bias and two parameters, both *w*_m_ and *w*_r_, are responsible for standard deviation. Previous studies also reported that Bayesian models with the Bayes least-squares strategy in the estimation stage described behavioral data in temporal reproduction task with hand response (Jazayeri and Shadlen, 2010; Acerbi et al., 2012). Our results supported these studies and extended these findings to sensory timing and saccadic eye movement. However, as we discussed earlier the Bayesian models are not the only way to investigate temporal computations. Future studies can compare different computational models of interval timing across different behavioral contexts.

## Data availability statement

The raw data supporting the conclusions of this article will be made available by the authors, without undue reservation.

## Ethics statement

The studies involving humans were approved by SCS Ethics Committee (IPM), School of Cognitive Sciences, Institute for Research in Fundamental Sciences. The studies were conducted in accordance with the local legislation and institutional requirements. The participants provided their written informed consent to participate in this study.

## Author contributions

AP and MS designed research, analyzed data, and wrote the paper. AP performed research. All authors contributed to the article and approved the submitted version.

## Conflict of interest

The authors declare that the research was conducted in the absence of any commercial or financial relationships that could be construed as a potential conflict of interest.

## Publisher’s note

All claims expressed in this article are solely those of the authors and do not necessarily represent those of their affiliated organizations, or those of the publisher, the editors and the reviewers. Any product that may be evaluated in this article, or claim that may be made by its manufacturer, is not guaranteed or endorsed by the publisher.
